# Development of Sesamol Carbamate-L-Phenylalanine Prodrug Targeting L-Type Amino Acid Transporter1 (LAT1) as a Potential Antiproliferative Agent against Melanoma

**DOI:** 10.3390/ijms23158446

**Published:** 2022-07-30

**Authors:** Tarapong Srisongkram, Katayun Bahrami, Juulia Järvinen, Juri Timonen, Jarkko Rautio, Natthida Weerapreeyakul

**Affiliations:** 1 Division of Pharmaceutical Chemistry, Faculty of Pharmaceutical Sciences, Khon Kaen University, Khon Kaen 40002, Thailand; tarasri@kku.ac.th; 2 Research Institute for Human High Performance and Health Promotion, Khon Kaen University, Khon Kaen 40002, Thailand; 3 School of Pharmacy, University of Eastern Finland, 70211 Kuopio, Finland; katayun.bahrami@uef.fi (K.B.); juulia.jarvinen@uef.fi (J.J.); juri.timonen@uef.fi (J.T.)

**Keywords:** sesamol, sesamol prodrug, L-phenylalanine, LAT1-mediated drug delivery, melanoma, LAT1 prodrug

## Abstract

Sesamol is a compound reported to have anti-melanogenesis and anti-melanoma actions. Sesamol, however, has low intracellular drug concentration and fast excretion, which can limit its benefits in the clinic. To overcome this drawback and increase intracellular delivery of sesamol into the target melanoma, research has focused on L-type amino acid transporter 1 (LAT1)-mediated prodrug delivery into melanoma cells. The sesamol prodrug was designed by conjugating sesamol with L-phenylalanine at the para position with a carbamate bond. LAT1 targeting was evaluated vis-à-vis a competitive [^14^C]-leucine uptake inhibition. The sesamol prodrug has a higher [^14^C]-leucine uptake inhibition than sesamol in human LAT1-transfected HEK293 cells. Moreover, the sesamol prodrug was taken up by LAT1-mediated transport into SK-MEL-2 cells more effectively than sesamol. The sesamol prodrug underwent complete hydrolysis, releasing the active sesamol at 72 h, which significantly exerted its cytotoxicity (IC_50_ of 29.3 µM) against SK-MEL-cells more than sesamol alone. Taken together, the strategy for LAT1-mediated prodrug delivery has utility for the selective uptake of sesamol, thereby increasing its intracellular concentration and antiproliferation activity, targeting melanoma SK-MEL-2 cells that overexpress the LAT1 protein. The sesamol prodrug thus warrants further evaluation in an in vivo model.

## 1. Introduction

Sesamol is an herbal medicine made from *Sesame indicum* Linn. Sesamol can induce apoptotic cell death in various cancer cell types, including colon cancer [1], adenocarcinoma lung cancer [2], malignant melanoma [3], and hepatocellular carcinoma [4]. Sesamol possesses selective antiproliferation in colon cancer [1], non-small cell lung cancer [5], and malignant melanoma [6] compared to non-cancerous Vero cells. Sesamol is considered as a chemotherapeutic molecule; however, the somewhat high solubility, low lipophilicity, and fast release of sesamol limits its transport across the cell membrane [6], requiring a high concentration in the extracellular compartment in order to exert its intracellular bioactivity [7]. This inefficiency occurs because sesamol has a low intracellular distribution in the target cells (i.e., malignant melanoma), and the intracellular concentration of sesamol is correlated with its antiproliferative activity [7,8]. It thus needed a higher concentration in the extracellular compartment to achieve antiproliferative activity and an inhibition concentration of 50% (IC_50_) [1,5,6]. Consequently, to overcome the low intracellular concentration of sesamol, permeation-enhancing approaches targeting cancer cells should be utilized. In addition, sesamol has been found to form a dimer during the oxidation process of oils [9]. Sesamol dimers have various low antioxidants, which can hinder sesamol’s bioactivities [10]. Preventing unwanted dimerization before intracellular delivery into the target cells is also important for sesamol to exert its bioactivity.

Malignant melanoma is one of the diseases that responds to sesamol treatment [3,6,11]. Sesamol has shown a role in anti-melanogenesis [12,13], antiproliferation against malignant melanoma [3,6,11], and skin protection [14]. Sesamol was found to induce apoptotic proteins (i.e., Bcl-2 and Bax) that are effective against malignant melanoma [3]. Sesamol uptake is associated with the L-type amino acid transporter 1 (LAT1), which is overexpressed in malignant melanoma [6]. Malignant melanoma has multiple mutations on several oncogenes that are associated with cell proliferation (viz., BRAF, NRAS, p53, CDK4, and PI3K genes) [15] and have been shown to have high nutrient transporter expression, including LAT1 and ASCT2 amino acid transporter [16] and GLUT1 glucose transporter [17]. Among the overexpressed transporters, the LAT1 protein has the highest overexpression in primary melanoma and malignant melanoma cell lines, including SK-MEL-2 [6], C8161, and 451Lu [18]. LAT1 is associated with disease progression and severity of malignant melanoma, which underscores the central role of LAT1 in melanoma cell growth [19]. LAT1 has lower expression in non-cancerous cells than in cancer cells [6,20]. The overexpression of LAT1 in melanoma cells over non-cancerous cells is useful for the targeted delivery of xenobiotic compounds into the melanoma cells or other high LAT1-expressing cancer cells.

The LAT1 targeting delivery system has been developed using a prodrug approach to transport several drug molecules into LAT1-overexpressed cells (i.e., blood–brain barrier or blood–retina barrier, or cancer cells) [20]. The prodrug approach is a chemical modification that makes the drug molecules inactive in non-target environments but activates the molecules in the target site through enzymatically catalyzed mechanism(s) [21]. Gabapentin and L-dopa are examples of molecules that use LAT1 to cross the blood–brain barrier [22]. Melphalan and acivicin are chemotherapeutic agents known to be transported via the LAT1 protein [20]. Chlorambucil conjugated with L-tyrosine is a successful example of prodrug development that targeted LAT1 to increase intracellular uptake and selectively transport it into cancer cells [23]. Thus, LAT1-mediated prodrug delivery is a promising mechanism to enhance both intracellular uptake and the antiproliferation effect. To date, there has been no report on using the prodrug approach for LAT1-mediated delivery of melanoma treatment molecules; hence, this study aimed to demonstrate and apply the LAT1-mediated delivery prodrug approach to improve intracellular sesamol uptake and, as a consequence, antiproliferation against melanoma cells.

LAT1-targeted prodrugs can be formed using hydrolysable bonds, such as ester, carbamate, and amide bonds [23,24,25]. Compared to the ester bond, the amide bond is more stable for chlorambucil conjugated with L-tyrosine [23]. Carbamate bonds (urethanes) have also provided good chemical stability for prodrug molecules [26]. Carbamate bonds bypass the first-pass effect, allowing prodrug distribution from circulation to the target site where the prodrug undergoes systemic hydrolysis to release the active drug [27]. In general, carbamate is an ester–amide combination, providing proteolytic stability as it serves as a peptide bond surrogate for several molecules and offering permeation through the cell membrane. The carbamate bond in LAT1-mediated delivery of dopamine has good stability in the systemic circulatory system [25]. Based on the sesamol structure having hydroxyl groups, sesamol can form a carbamate bond with LAT1 substrates.

In the current study, L-phenylalanine was coupled to sesamol, as it is a natural LAT1 substrate that provides a high susceptibility to the LAT1 transporter protein and is regarded as safe in the physiological system. L-phenylalanine is one of several compounds that possesses a high LAT1 susceptibility that inhibits [^3^H] gabapentin uptake by human LAT1 (hLAT1)-transfected HEK293 cells (IC_50_ of 69 ± 29 µM) [28]. Previous studies showed that substitution at the para position of the phenyl ring of L-phenylalanine provided a high susceptibility to the LAT1 protein. For example, para-OH-L-phenylalanine inhibited [^3^H]-gabapentin uptake in hLAT1-HEK293 cells (IC_50_ of 68 µM) [28], and para-valproic acid conjugated with L-phenylalanine inhibited [^14^C]-leucine in breast cancer (IC_50_ of 40 µM) [24]. In the present study, the carbamate linker formed at the para position of L-phenylalanine with the hydroxyl group of sesamol. The LAT1 susceptibility of sesamol coupled with L-phenylalanine or the sesamol prodrug was evaluated by competitive [^14^C]-leucine uptake inhibition in hLAT1-HEK293 cells. Since the human malignant melanoma SK-MEL-2 cells are a considerably more clinically relevant model because they express high levels of the LAT1 protein [6], cellular uptake, metabolic conversion, and cytotoxicity of the sesamol prodrug were compared with sesamol.

## 2. Results

### 2.1. Synthesis Reaction and Chemical Characterization

**Figure 1:**
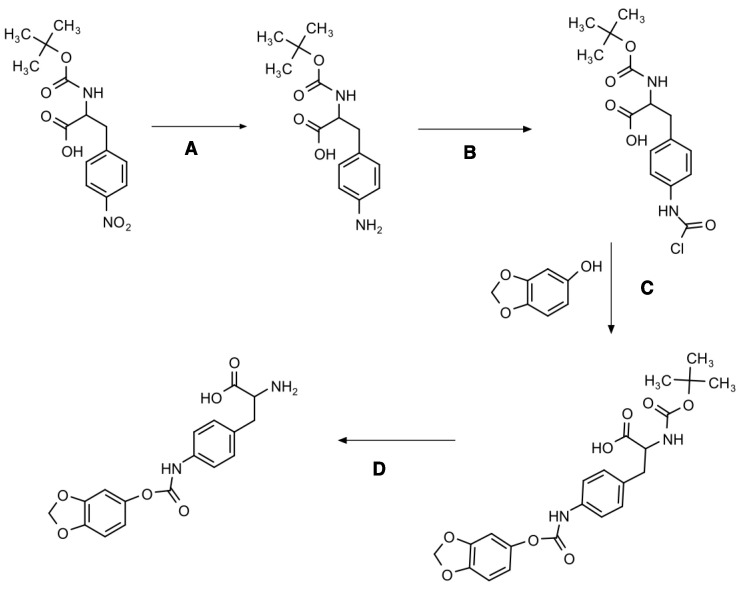
Synthesis diagram of 4-sesamol carbamate-L-phenylalanine prodrug with four multi-step reactions. Reagent and conditions: (**A**) Palladium, methanol, H_2_ gas, overnight; (**B**) triphosgene, triethylamine, anhydrous DCM, 1 h; (**C**) sesamol, triethylamine, tetrahydrofuran, overnight; and (**D**) trifluoracetic acid, anhydrous DCM, 4 h.

Figure 1 and Figures S1–S6 present the chemical synthesis and the chemical elucidation of the sesamol prodrug. The synthesis of 4-sesamol carbamate-L-phenylalanine prodrug (labelled as sesamol prodrug) was done within four reactions, as shown in Figure 1. The chemical elucidation was done by ^1^H-, ^13^C-, COSY-, HSQC-NMR, and ESI-MS positive (Figures S2–S6, respectively). The sesamol prodrug structure is illustrated in reaction D of Figure 1 and Figure S1. The chemical purity as analyzed by quantitative HNMR was 98.66%.

### 2.2. [^14^C]-Leucine Uptake Inhibition

**Figure 2:**
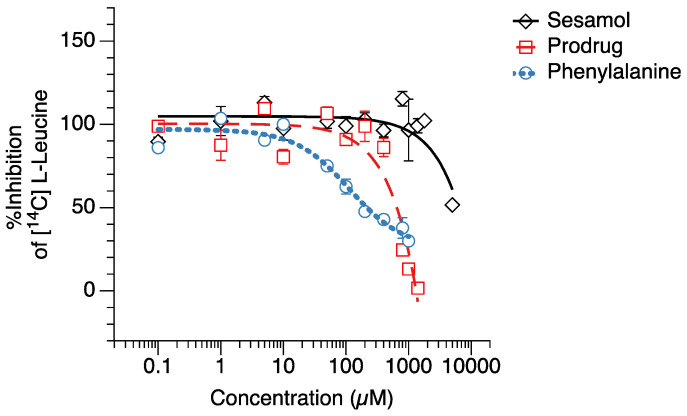
[^14^C]-Leucine uptake inhibition testing of (◇) sesamol, (☐) sesamol prodrug, and (○) L-phenylalanine in hLAT1-transfected HEK293 cells.

The competitive [^14^C]-leucine uptake inhibition of the compounds (prodrug, sesamol, and L-phenylalanine) was tested in the hLAT1-HEK293 cells (Figure 2). The hLAT1-HEK293 cells were used in competitive [^14^C]-leucine uptake inhibition testing because they provide the selective expression of the LAT1 transporter compared to other cell types that may have several influx transporters expressed in the cell membrane [29]. We found that the sesamol prodrug had a significantly lower IC_50_ value (631.3 ± 38.0 µM) than L-phenylalanine (96.8 ± 11.5 µM) but a significantly higher IC_50_ value than sesamol itself (4755.4 ± 218.8 µM) (Table 1). This result suggests that the sesamol prodrug can inhibit LAT1 in transfected human cells stronger than sesamol but weaker than its natural substrate. 

### 2.3. Cellular Uptake in Melanoma Cells

**Figure 3:**
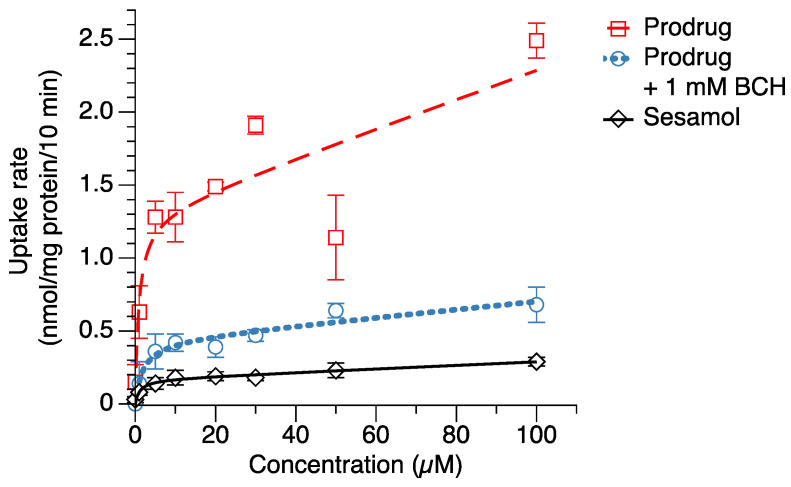
Cellular uptake of sesamol (◇), sesamol prodrug (☐), and sesamol prodrug co-incubated with 1 mM BCH (○) in melanoma SK-MEL-2 cells. The uptake rates were fitted with a nonlinear least-squares kinetics model and are represented as dash line (--), dot line (···), and straight line (−) for prodrug, prodrug with 1 mM BCH, and sesamol uptakes, respectively. Data are expressed as mean ± standard deviation of three replicates.

In order to evaluate the intracellular delivery of the sesamol prodrug in target cells, the cellular uptake of the sesamol prodrug and sesamol in melanoma SK-MEL-2 cells was determined. The results showed that the sesamol prodrug and sesamol were taken up via both saturated (carrier-mediated transport) and non-saturated (passive transport) mechanisms (Figure 3). The uptake parameters of the sesamol prodrug and sesamol in saturated (carrier-mediated) and non-saturated (passive) transport are presented in Table 2. The maximum carrier-mediated transport uptake velocity (V_max_) of the sesamol prodrug was 1.3 ± 0.2 nmol/mg protein/10 min, which was 6.5 times greater than the V_max_ of sesamol (0.2 ± 0.1 nmol/mg protein/10 min) (*p* < 0.05). These results suggest that the prodrug has a higher carrier-mediated uptake rate than sesamol. The compound concentration that causes half of V_max_ (K_m_) of the sesamol prodrug was 1.0 ± 0.7 µM, comparable with the K_m_ of sesamol (1.1 ± 0.5 µM). The K_m_ value indicates the compound concentration at the half maximum velocity. A lower K_m_ indicates a higher susceptibility of the compound to carrier-mediated transport. Our result suggests that the sesamol prodrug has a susceptibility to carrier-mediated transport similar to sesamol. The passive transport uptake rate (P_d_) of the sesamol prodrug was 9.8 ± 0.7 µL/mg protein/10 min, which was 24.5 times greater than that of sesamol (1.3 ± 0.6 µL/mg protein/10 min) (*p* < 0.05). The results suggest that the sesamol prodrug also has higher passive transport than the sesamol. The higher passive transports of the sesamol product may came from the uptake mechanism of the sesamol prodrug by the LAT1 carrier-mediated transporter. The LAT1 transporter is the facilitated diffusion or passive transport transporter [30]; thereby, we can observe a combination of carrier-mediated transport and passive transport of the sesamol prodrug.

In general, the LAT1 protein is a solute carrier that facilitates the transport of its substrates into the cells without energy consumption [31]. The LAT1 substrates (i.e., gabapentin or pregabalin) have both carrier-mediated transport and passive transport [32,33]. The physicochemical properties of sesamol conjugated with L-phenylalanine also changed from the sesamol alone. Sesamol has a lipophilicity (logP and logD_7.4_) equal to 1.29, and a polar surface area (PSA) of 38.7, whereas sesamol conjugated with L-phenylalanine had a logP of 2.22, a logD_7.4_ of −0.05, and a PSA of 120.11 (calculated by MarvinSketch version 20.19, ChemAxon, Budapest, Hungary). The low logD_7.4_ of the sesamol prodrug indicates the low possibility of the compound to be taken up by membrane passive transports. However, our result shows the high passive transports from the sesamol prodrug, which indicates that the compound has been facilitated by the LAT1-facilitated diffusion transport. The total cellular uptake of the sesamol prodrug could, therefore, result from both carrier-mediated and passive transport of the LAT1 protein in melanoma cells.

When the sesamol prodrug was co-incubated with 1 mM BCH (LAT1 inhibitor) (Figure 3), the V_max_ of the prodrug with BCH was 3.3 times less than the prodrug alone (V_max_ = 0.4 ± 0.1 nmol/mg protein/10 min). The K_m_ of the prodrug with BCH was 1.7 times greater than the prodrug alone (K_m_ = 1.7 ± 1.4 µM), a non-significant difference. The reduction in V_max_ and K_m_ when co-incubated with BCH indicates that the sesamol prodrug was taken up by the LAT1-mediated transport. The P_d_ of the prodrug with BCH was also 3.5 times less than the prodrug alone (P_d_ = 2.8 ± 0.7 µL/mg protein/10 min) (*p* < 0.05). The reduction in P_d_ when co-incubated with the LAT1 inhibitor indicates that the passive transport of the sesamol prodrug came from the LAT1 protein. The results demonstrate the role of LAT1 as a solute carrier transport that facilitated substrate uptake via the concentration gradient. This result confirms that the sesamol prodrug was taken up via LAT1.

### 2.4. In Vitro Stability Testing

**Figure 4:**
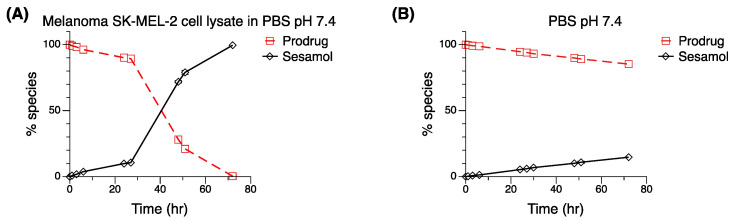
In vitro stability testing of sesamol prodrug (☐) and its parent compound—sesamol (◇). An amount of 10 µM of sesamol prodrug dissolved in (**A**) SK-MEL-2 lysate in PBS pH 7.4 compared with 10 µM of sesamol prodrug dissolved in (**B**) PBS pH 7.4. Standard deviations were low: error bars smaller than the symbols.

In vitro stability of the sesamol prodrug was evaluated under two incubation conditions at 37 °C (Figure 4), which were in the SK-MEL-2 lysate in PBS pH 7.4 (Figure 4A) and PBS pH 7.4 (Figure 4B). At 72 h in PBS pH 7.4, the sesamol prodrug was still intact, with 85.3% remaining, indicating the stability of the sesamol prodrug under pH 7.4 phosphate-buffered solution. Following the reaction for 75 h, the sesamol prodrug was completely hydrolyzed into free sesamol at 72 h in the SK-MEL-2 cell lysate, with only 0.4% of the sesamol prodrug remaining. The result suggests that the sesamol prodrug was activated in the melanoma cells to release sesamol. Notwithstanding, the fast excretion of sesamol in an aqueous solution is one of the obstacles that have retarded the use of sesamol in a clinical setting [34,35]. The fast excretion could be solved because we found that the sesamol prodrug remained for a long period before being completely hydrolyzed into the sesamol (72 h). There could be further opportunity to explore whether the systemic circulation of the sesamol prodrug is improved compared to sesamol alone.

### 2.5. Cytotoxicity of Sesamol Prodrug

**Figure 5:**
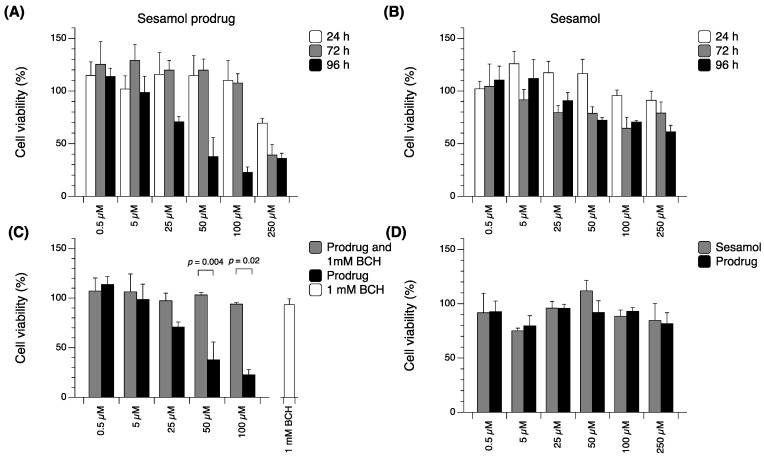
Cytotoxicity of (**A**) sesamol prodrug and (**B**) sesamol in SK-MEL-2 cells at various times. (**C**) Cytotoxicity of sesamol prodrug compared with sesamol prodrug with 1 mM BCH in SK-MEL-2 cells. (**D**) Cytotoxicity of sesamol prodrug compared with sesamol at 96 h in Vero cells. Data are expressed as means ± standard deviations of three replicates. A *p*-value less than 0.05 is statistically significant.

Cytotoxicity against melanoma cells was evaluated as the pharmacological response of the sesamol prodrug (Figure 5). The results showed that the sesamol prodrug and sesamol could reduce the proliferation of melanoma cells in a time- and concentration-dependent manner (Figure 5A,B). The sesamol prodrug was active at 96 h and showed cytotoxicity (IC_50_ of 29.3 ± 0.8 µM), with a maximum effect (E_max_) at 29.8 ± 2.0% (Figure 5A and Table 3). Sesamol started to exert cytotoxicity earlier at 72 h, but with an E_max_ of 250 µM at 60.6 ± 11.7% (Figure 5B and Table 3). When comparing the cytotoxicity between the sesamol prodrug and sesamol at 96 h, the cytotoxicity of the sesamol prodrug was significantly greater at a concentration 100 µM (*p* < 0.05) (Figure 5A,B). Hence, the sesamol prodrug is more effective than sesamol, and the prodrug also provides a sustained release effect. The cytotoxicity of the sesamol prodrug was significantly inhibited by 1 mM BCH (*p* < 0.05) (Figure 5C), confirming that the sesamol prodrug was taken up via LAT1 in melanoma SK-MEL-2. The cytotoxicity of the prodrug against Vero cells was also performed to demonstrate the safety of the sesamol prodrug in non-cancerous cells (Figure 5D). The results showed that the sesamol prodrug and sesamol exhibited no significant cytotoxic to non-cancerous Vero cells at 96 h. This result confirms the safety of the sesamol prodrug in non-cancerous cells. However, care must be taken when considering other high LAT1-expressing tissues, such as blood–brain barrier (BBB), placenta, and testis, because the sesamol prodrug may have effects in those tissues due to the rich LAT1 expression.

## 3. Discussion

Herbal medicines are recognized as having advantages for numerous reasons, including availability, reasonable price, good efficacy with high selectivity, and low toxicity [36]. Sesamol is a natural compound that possesses several advantages for human health, including antioxidation, anti-inflammation, reduction in cholesterol in the bloodstream, skin protection as anti-aging, and anti-cancer activity against several cancer types with less damage to non-cancerous cells [37].

Previous studies have shown that the LAT1 protein played a pivotal role in the carrier-mediated transport of sesamol uptake and its selective cytotoxicity in the high LAT1 mRNA expression melanoma SK-MEL-2 cells compared to low LAT1 mRNA expression non-cancerous Vero cells [6]. However, the structure of sesamol does not fully comply to the LAT1 pocket site. Therefore, it does not provide a significant intracellular concentration to exert the high cytotoxicity from the sesamol. This study provides a better insight into the role of LAT1 in the sesamol uptake in the hLAT1-HEK293 cells and also demonstrates that the chemical modification prodrug strategy of sesamol with a LAT1 substrate could overcome the low intracellular concentration of sesamol and increase its cellular potency in melanoma cells.

Previous studies showed that a high sesamol concentration at the extracellular compartment was required to achieve the cytotoxic effect. The reported IC_50_ values of sesamol against SK-MEL-2, SK-LU-2, and HCT-116 are 2 – 3 mM [5,6,11], 2.0 mM [2], and 2.6 mM [1]. With a LAT1-mediated delivery system, the IC_50_ value of the sesamol prodrug in SK-MEL-2 cells was reduced to 29.3 µM, which is 60 times more potent than sesamol alone. This phenomenon may relate to the intracellular concentration of sesamol (=V_max_/K_m_ + P_d_), which also increased from 1.48 µL/mg protein/10 min to 11.1 µL/mg protein/10 min (7.5 times higher than sesamol) when it was in a prodrug form. Abdelhamid et al. reported that encapsulation of sesamol into cadmium sulfide quantum nanoparticle dots exerted an IC_50_ of 847.8 µM in MCF-7 cells at 1730 µg/mL [34]. Geetha, Kapila, et al. reported that sesamol-loaded nanoparticles with 10 to 100 µg of sesamol inhibited ML-60 and Molt-4 cell lines [38]. Sesamol conjugated with PEG-selenium nanoparticles inhibited HepG2 cells with an IC_50_ value of 68.7 µg/mL [39]. The IC_50_ of the sesamol prodrug was lower compared to the IC_50_ of melphalan (IC_50_ = 0.1 ± 0.0 mM) against melanoma cells [6]. Our data confirm that sesamol conjugated with L-phenylalanine for LAT1-mediated intracellular uptake has potential as an efficient strategy. LAT1 targeting by the sesamol prodrug showed very high cytotoxicity against melanoma cells compared to its cytotoxicity in other cell lines.

The role of carrier-mediated transport and passive transport can be identified using cellular uptake parameters, which are V_max_, K_m_, and P_d_ [32]. The total uptake clearance of the sesamol prodrug by carrier-mediated transport (V_max_/K_m_) was 1.3 mL/mg protein/10 min, and by passive transport (P_d_) was 9.8 µL/mg protein/10 min or 0.0098 mL/mg protein/10 min. Thus, the total uptake clearance of passive transport was 132.7 times lower than carrier-mediated transport of the sesamol prodrug. This difference can be confirmed by using the inhibition concentration at 50% (IC_50_) of the sesamol prodrug in melanoma to calculate the total uptake velocity for each transportation route. At an IC_50_ (29.3 µM), the carrier-mediated transport velocity (=V_max_ × [S])/(K_m_ + [S]) will be 1.3 nmol/mg protein/10 min, and the passive transport velocity (P_d_ × [S]) will be 0.29 nmol/mg protein/10 min, which is 4.5 times lower than carrier-mediated transport. This evidence suggests that carrier-mediated transport of the prodrug has a higher role in prodrug uptake than passive transport. The sesamol prodrug’s [^14^C]-leucine uptake inhibition in hLAT1-HEK293 cells and the reduction in cellular uptake and cytotoxicity of the prodrug in the presence of BCH (a LAT1 inhibitor) confirm that LAT1-mediated transport of the sesamol prodrug governs the uptake transportation route of the sesamol prodrug across the cell membrane.

The conjugation with L-phenylalanine via carbamate linkage provided a sustained release of sesamol from the prodrug. The sesamol prodrug was completely cleaved after 72 h in SK-MEL-2 lysate, which is correlated to the prodrug’s cytotoxicity being exerted after completing 96 h of treatment. The onset of cytotoxicity activity of the prodrug was reasonably associated with the hydrolysis of the carbamate linkage with a 24 h time gap between the prodrug hydrolysis and its activity being exerted. Since the sesamol prodrug was still intact in the aqueous system without cellular enzymes (PBS pH 7.4) after 72 h, it is evident that the release of sesamol from the prodrug occurred via enzymatic hydrolysis in the cells. The carbamate linkage can be hydrolyzed by plasma cholinesterase, CYP450, and esterase enzymes [40]. These esterases are found in the plasma, liver, and skin cells [41]. The expression of esterases also affected the pharmacological response of the prodrug molecules [42]: the latter requires further study to clarify the role of esterases in enzymatic hydrolysis of the sesamol prodrug. Taken together, the carbamate linkage provides the ability to prolong the duration of action of sesamol, which can be beneficial for therapeutic outcomes because of less frequent administration and a lower cost.

## 4. Materials and Methods

### 4.1. Materials

Boc-4-nitro-L-phenylalanine (99.77% purity) was obtained from Chem Scene L.S. (Monmouth Junction, NJ, USA). Sesamol (98% purity) and triphosgene (99% purity) were obtained from Acros organics (New York, NY, USA). Palladium on 10% activated charcoal, L-phenylalanine, and cellite were obtained from Sigma-Aldrich (St. Louis, MO, USA). Tetrahydrofuran and triethylamine (99% purity) were obtained from Merck-Schuchardt-OHG (Hohenbrunn, Germany). Dichloromethane (DCM) (99.9% purity), Acetonitrile (99.9% purity) and formic acid (98% purity) were obtained from Honeywell Riedel-de-Haën™ (Seelze, Germany). Trifluoracetic acid (99.5% purity) was obtained from Apollo Scientific Limited (Manchester, UK). The culture media, Dulbecco’s modified Eagle’s medium of high glucose (DMEM), reagents such as Hank’s balanced salt solution (HBSS) without Ca^2+^ and Mg^2+^ supplement, and penicillin and streptomycin were purchased from GIBCO, Invitrogen (Grand Island, NY, USA). Fetal bovine serum (FBS) was purchased from G.E. Life Sciences (Parramatta, Australia).

### 4.2. General Synthesis Procedure

Thin-layer chromatography (TLC) silica gel 60 F_254_ (Merck Millipore, Darmstadt, Germany) was used for monitoring synthesis reactions. Nuclear magnetic resonance (NMR) spectra were measured by a Bruker Ascend^TM^ 600 MHz spectrometer (Bruker, Fällanden, Switzerland). The NMR measurements for ^1^H and ^13^C were made using 600.18 MHz and 150.91 MHz, respectively, with tetramethylsilane as an internal standard. The intermediate product was purified with flash chromatography (Buchi), while the final product was purified by preparative high-performance liquid chromatography (HPLC) with the resin-based Supelcogel^TM^ ODP-50 (25 cm × 21.2 mm, 5 µm) from Sigma-Aldrich (St. Louis, MO, USA). The purity of the final product was calculated by quantitative ^1^H NMR using maleic acid as an internal standard. Mass spectra were measured by a Finnigan^TM^ LTQ^TM^ Mass spectrometer (Thermo Fisher Scientific, Waltham, MA, USA).

### 4.3. Synthesis of Sesamol Phenylalanine Carbamate Prodrug

#### 4.3.1. Boc-4-Amino-L-Phenylalanine Synthesis (A)

Boc-4-nitro-L-phenylalanine (3.52 g) and palladium (0.1 g) were carefully dissolved in methanol. The reaction mixture was filled with hydrogen gas (4 bar) and stirred overnight. The reaction mixture was filtered through cellite and dried under reduced pressure, yielding the solid orange product of Boc-4-amino-L-phenylalanine (3.01 g, 90.2% yield). ^1^H NMR (CDCl_3_, 600.18 MHz) δ ppm: 1.42 (s, 9H), 3.02 (s, 2H), 4.48 (s, 4.9H), 4.96 (d, 1H, J = 6.97 Hz), 6.62–6.64 (d, 2H, J = 6.85 Hz), 6.95–6.97 (d, 2H, J = 8.19 Hz). The product was used without further purification.

#### 4.3.2. Carbamic Chloride Formation of Boc-4-Amino-L-Phenylalanine (B)

Triphosgene (0.62 g) was dissolved in cold anhydrous DCM. Boc-4-amino-L-phenylalanine (1.52 g) and triethylamine (1.5 mL) were dissolved in anhydrous DCM, added dropwise into triphosgene solution, and stirred for 40 min on ice. The temperature was increased to room temperature, and the reaction was stirred for 2 h. The reaction was monitored using TLC (methanol/DCM). Then, the reaction mixture was evaporated and dried under reduced pressure to obtain a yellow mixture product of Boc-4-chloroformate-L-phenylalanine. ^1^H NMR (CDCl_3_, 600.18 MHz) δ ppm: 1.4 (s, 41H), 3.11 (s, 18H), 4.54 (d, 1H, J = 6.70 Hz), 4.97 (d, 1H, J = 8.08 Hz), 7.0 (d, 2H, J = 8.00 Hz), 7.14 (d, 2H, J = 8.35 Hz,), 11.8 (s, 3H). The product was used without further purification.

#### 4.3.3. Sesamol Conjugation (C)

Boc-4-carbamate-L-phenylalanine was dissolved in cold tetrahydrofuran. Sesamol (0.72 g) and triethylamine (1.5 mL) were dissolved in THF and added dropwise into the Boc-4-carbamate-L-phenylalanine solution and stirred for 40 min on ice. The temperature was increased to room temperature and incubated overnight. Then, the reaction mixture was evaporated and purified twice by 40 g silica flash chromatography (round 1: gradient elution of methanol and dichloromethane; round 2: gradient elution of ethyl acetate and petroleum ether) to obtain a white solid product—Boc-4-sesamol carbamate-L-phenylalanine (0.4 g, 16.7% yield). ^1^H NMR (CDCl_3_, 600.18 MHz) δ ppm: 1.43 (s, 10H), 3.12 (s, 3H), 3.78 (s, 1H), 4.58 (s, 1H), 4.94 (s, 1H), 5.98 (s, 2H), 6.60 (dd, 1H, J = 8.41 Hz), 6.69 (d, 1H, J = 2.31 Hz), 6.76 (d, 1H, J = 8.41 Hz), 7.12 (d, 2H, J = 8.45 Hz), 7.39 (d, 2H, J = 6.75 Hz). The product was used without further purification

#### 4.3.4. Hydrolysis of Boc Protection Group (D)

Boc-4-sesamol carbamate-L-phenylalanine (340 mg) was dissolved in cold DCM. Trifluoroacetic acid (TFA) solution (1 mL) was added dropwise and stirred for 30 min on ice. The temperature was increased to room temperature and stirred for 4 h. Then, the reaction mixture was evaporated under an evaporator and high vacuum. The DCM was added and evaporated again until it was completely dried to obtain a white solid product of 4-sesamol carbamate-L-phenylalanine (280 mg). Then, the product was purified using preparative UV-HPLC with the resin-based Supelcogel^TM^ ODP-50 (25 cm × 21.2 mm, 5 µm) column and the isocratic 20% acetonitrile in 0.1% formic acid as the mobile phase (10 mL/min). The sesamol prodrug was eluted at 17 min with the dry weight of 68.3 mg (26.1 % yield). The purity of the product was 98.66% (determined using quantitative proton NMR). ^1^H NMR (MeOD-d_4_, 600.18 MHz) δ ppm: 3.13–3.31 (m, 2H), 4.24 (dd, 1H, J = 5.42, 7.61 Hz), 6.00 (s, 2H), 6.63–6.65 (dd, 1H, J = 2.37, 8.42 Hz), 6.73–6.74 (d, 1H, J = 2.23 Hz), 6.82–6.83 (d, 1H, J = 8.48 Hz), 7.27–7.28 (d, 2H, J = 8.97 Hz), 7.51–7.52 (d, 2H, J = 8.97 Hz); and ^13^C NMR (MeOD-d_4_, 150.91 MHz): 35.30 (1 × C), 48.16 (1 × C), 53.71 (1 × C), 101.72 (1 × C), 103.50 (1 × C), 107.35 (1 × C), 113.87 (1 × C), 119.16 (2 × C), 128.96 (1 × C), 129.65 (2 × C), 138.15 (1 × C), 145.18 (1 × C), 145.30 (1 × C), 148.03 (1 × C), 153.09 (1 × C), 169.80 (1 × C). MS (m/z): [M]^+^ calculated for C_17_H_16_N_2_O_6_ 344.32, found 344.95 in 0.1% formic acid in methanol with positive mode electron spray ionization (ESI).

### 4.4. Cell Culture

Human LAT1 (SLC7A5) transfected in Flp-In™-293 human embryonic kidney (HEK293) cells (R750-07, Invitrogen, CA, USA), African green monkey kidney epithelial Vero cell (ATCC#CCL-81), and human melanoma SK-MEL-2 cells (CLS-Cell lines Service, Eppelheim, Germany) were maintained in DMEM with 10% FBS, 100 unit/mL of penicillin, and 100 µg/mL of streptomycin at 37 °C in 5% CO_2_ atmosphere. L-Glutamine (2 mM) and Hygromycin B (12 µg/mL) were added for maintaining the hLAT1-HEK293 cells.

### 4.5. [^14^C]-Leucine Uptake Inhibition

The hLAT1-HEK293 cells (1 × 10^5^ cells/well) were incubated in a 24-well plate at 37 °C for 48 h. The cells were washed with pre-warm HBSS, co-incubated with the test compounds (0.1–1000 µM), and radiolabeled L-leucine for 10 min. The ice-cold HBSS was added to stop the reaction. The cells were washed twice with ice-cold HBSS and lysed in 0.1 M NaOH for 1 h. The liquid scintillation cocktail (Ultima Gold, Perkin Elmer, Waltham, MA, USA) was added, and the radiolabeled L-leucine was quantified using a scintillation counter (Wallac 1450 MicroBeta; Wallac Oy, Finland).

### 4.6. Cellular Uptake

The SK-MEL-2 cells (2 × 10^5^ cells/well) were incubated (37 °C) for 48 h in a 24-well plate. Then, the medium was removed, and the cells were pre-incubated with HBSS for 10 min. The cells were treated with compound (1–100 µM) for another 10 min. The ice-cold PBS was added, and the cells were washed twice with ice-cold PBS and solubilized in acetonitrile. Protein was removed by centrifugation at 2400× g at 4 °C for 20 min. The precipitated protein was re-solubilized by 0.1 M NaOH before protein determination. The compound was quantified using HPLC analysis 4.7. The uptake rates were fitted using a nonlinear least-squares kinetics model that allowed estimation of V_max_, K_m_, and P_d_ values [8].

### 4.7. HPLC Analysis

Quantitative analysis of sesamol and sesamol prodrug were performed using a Shimadzu HPLC (LC-2030C-3D) with UV photodiode array detector (Kyoto, Japan). The analysis was performed using a BDS Hypersil C-18 column (4.6 × 250 mm, 5 µm, Thermo Fisher Scientific, Waltham, MA, USA) with 30 °C column temperature and 0.8 mL/min flow rate. The mobile phase for sesamol was 70% acetonitrile in water, while the sesamol prodrug was 20% acetonitrile and 0.1% formic acid in water. The detection wavelength and elution time for the sesamol and sesamol prodrug were 292 nm and 240 nm, and 3.8 and 18.0 min, respectively. For metabolic stability, the mobile phase was 20% acetonitrile and 0.1% formic acid in water with detection wavelength at 292 nm. The HPLC analysis of sesamol was validated and optimized following the previous report [7]. The HPLC method for the sesamol prodrug was validated and yielded reliable analytical parameters with a limit of detection of 0.05 µM and a limit of quantification of 0.15 µM. The HPLC chromatograms are shown in supplementary Figure S7.

### 4.8. In Vitro Stability of Sesamol Prodrug

The sesamol prodrug (final concentration was 10 μM) was separately subjected to (1) PBS pH 7.4 and (2) SK-MEL-2 (4.5 × 10^6^ cells) homogenized cell lysate in PBS. The reaction was stirred (200 rpm) and incubated (37 °C) for 72 h. At each time point, the solution was pipetted out and injected into HPLC analysis. The HPLC analysis condition was used as described in the HPLC analysis 4.7.

### 4.9. Cytotoxicity of Sesamol and Sesamol Prodrug

The SK-MEL-2 and Vero cells were separately seeded into 96-well plates (5 × 10^4^ cells/well) for 48 h. The cells were incubated with 0.1–250 μM compound solution for another 48 h. Then, a neutral red solution (50 µg/mL) was added and incubated for 2 h. The cells were washed using PBS and lysed with 0.33% HCl in isopropanol. The absorbance of neutral red was measured at 540 nm using a UV-microplate reader (Perkin Elmer, Waltham, MA, USA). The % cell viability was calculated with respect to the control.

### 4.10. Statistical Analysis

All experimental results were expressed as means with a standard deviation of three replications. The nonlinear least-squares were performed using Datagraph 4.5 (Visual data tools Inc., Chapel Hill, NC, USA). The difference between samples of independent observations was analyzed using a Kruskal–Wallis nonparametric statistic in SPSS 24.0 (SPSS Inc., Chicago, IL, USA) and R software 4.2.1. The differences between samples with *p*-values less than 0.05 were considered statistically significant.

## 5. Conclusions

In summary, we report the strategy to improve sesamol intracellular uptake by using a LAT1-mediated prodrug delivery system. The hydroxyl group of sesamol was conjugated with L-phenylalanine at the para position via a carbamate bond. This sesamol prodrug has a high susceptibility to the LAT1 protein in hLAT1-HEK293 cells compared to sesamol alone. The sesamol prodrug was mostly taken up via the LAT1 protein and less so via passive transport. The LAT1 protein has a role in the selective cytotoxic effect of the sesamol prodrug by enhancing its cytotoxicity against the SK-MEL-2 cells, leading to its being non-cytotoxic to normal cells (Vero cells). The novel sesamol prodrug molecule has the potential to be an effective in vivo cytotoxic agent or clinically relevant in melanoma models. It may also be effective in other LAT1-overexpressed cancer cell types.

## Figures and Tables

**Table 1:** Half-maximal inhibitory concentration (IC_50_) of test compounds on [^14^C] L-leucine in hLAT1-HEK293 cells.

Compound	IC_50_ (µM)
L-Phenylalanine	96.8 ± 11.5
Sesamol	4755.4 ± 218.8
Sesamol prodrug	631.3 ± 38.0

**Table 2:** Uptake kinetic values of sesamol and sesamol prodrug in melanoma cells.

Compound	V_max_ (nmol/mg Protein/10 min)	K_m_ (µM)	P_d_ (µL/mg Protein/10 min)
Sesamol	0.2 ± 0.1	1.1 ± 0.6	1.3 ± 0.6
Sesamol prodrug	1.3 ± 0.2	1.0 ± 0.7	9.8 ± 0.7
Sesamol prodrug + 1 mM BCH	0.4 ± 0.1	1.7 ± 1.4	2.8 ± 0.7

**Table 3:** Cytotoxicity values of sesamol and sesamol prodrug at 0.5–250 µM in melanoma SK-MEL-2 cells at 96 h.

Compound	IC_50_ (µM)	E_max_ (%)
Sesamol	Not detected	60.6 ± 11.7
Sesamol prodrug	29.3 ± 0.8	29.8 ± 2.0

## Data Availability

Not applicable.

## Supplementary Materials

The following supporting information can be downloaded at: https://www.mdpi.com/article/10.3390/ijms23158446/s1.
