# Supplementary material for: Development of Sesamol Carbamate-L-Phenylalanine Prodrug Targeting L-Type Amino Acid Transporter1 (LAT1) as a Potential Antiproliferative Agent against Melanoma
Source: Int J Mol Sci. 2022 Jul 30;23(15):8446. doi: 10.3390/ijms23158446 (PMC9369069; doi:10.3390/ijms23158446)
Supplement: Supplementary file 1 [file ijms-23-08446-s001.zip › ijms-1839452-supplementary.pdf]

Supplementary Material

Development of sesamol carbamate-L-phenylalanine prodrug targeting L-type amino acid transporter1 (LAT1) as a potential antiproliferative agent against melanoma

Tarapong Srisongkram[1,2], Katayun Bahrami[3], Juulia Järvinen[3], Juri Timonen[3], Jarkko Rautio[3,*], Natthida Weerapreeyakul[1,2,*]

[1] Division of Pharmaceutical Chemistry, Faculty of Pharmaceutical Sciences, Khon Kaen University, Khon Kaen, 40002, Thailand; tarasri@kku.ac.th (T.S.); natthida@kku.ac.th (N.W.)
[2] Research Institute for Human High Performance and Health Promotion, Khon Kaen University, Khon Kaen, 40002, Thailand; tarasri@kku.ac.th (T.S.); natthida@kku.ac.th (N.W.)
[3] School of Pharmacy, University of Eastern Finland, Kuopio 70211, Finland; katayun.bahrami@uef.fi (K.B.), juulia.jarvinen@uef.fi (J.J.); juri.timonen@uef.fi (J.T.); jarkko.rautio@uef.fi (J.R.)

* Correspondence: natthida@kku.ac.th; jarkko.rautio@uef.fi

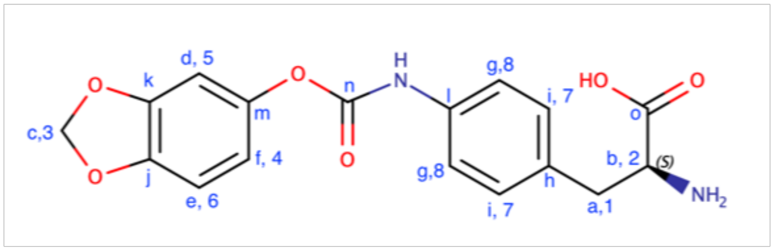

Figure S1. Sesamol prodrug chemical structure.

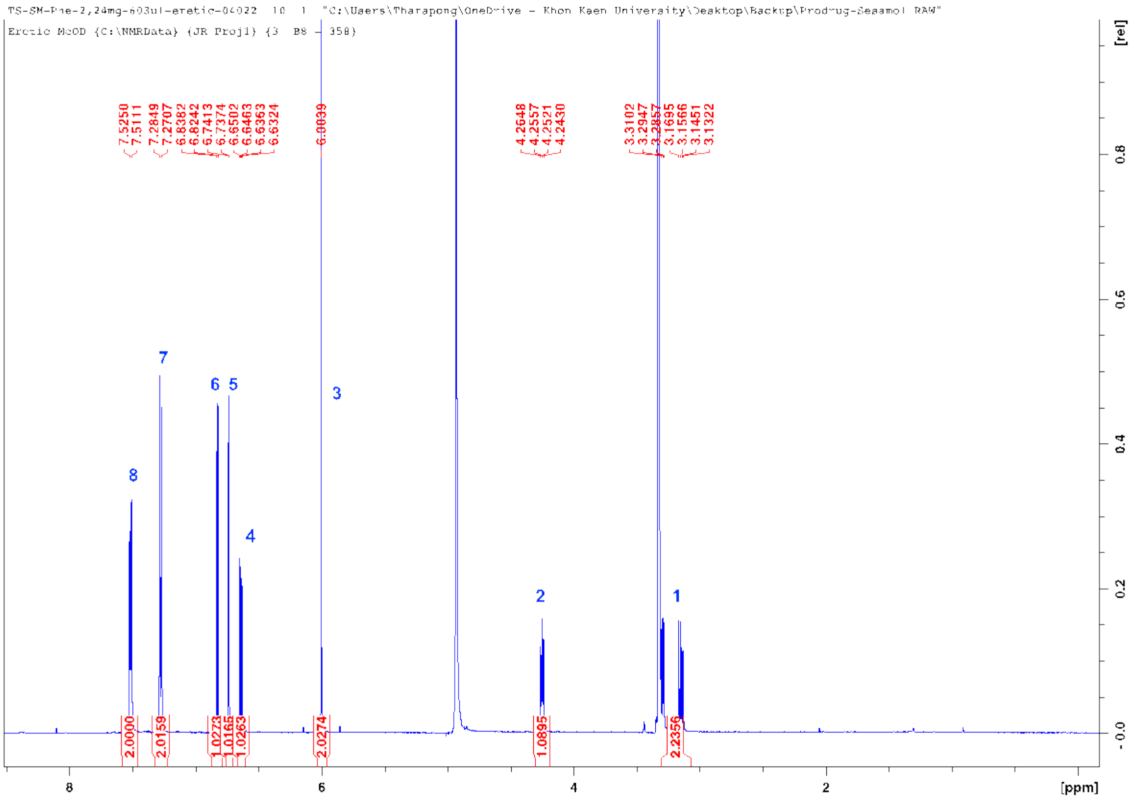

Figure S2. Proton (^{1}H) nuclear magnetic spectra (NMR) of sesamol prodrug in methanol-d solvent. The number **1-8** proton positions from the chemical structure from Figure S1

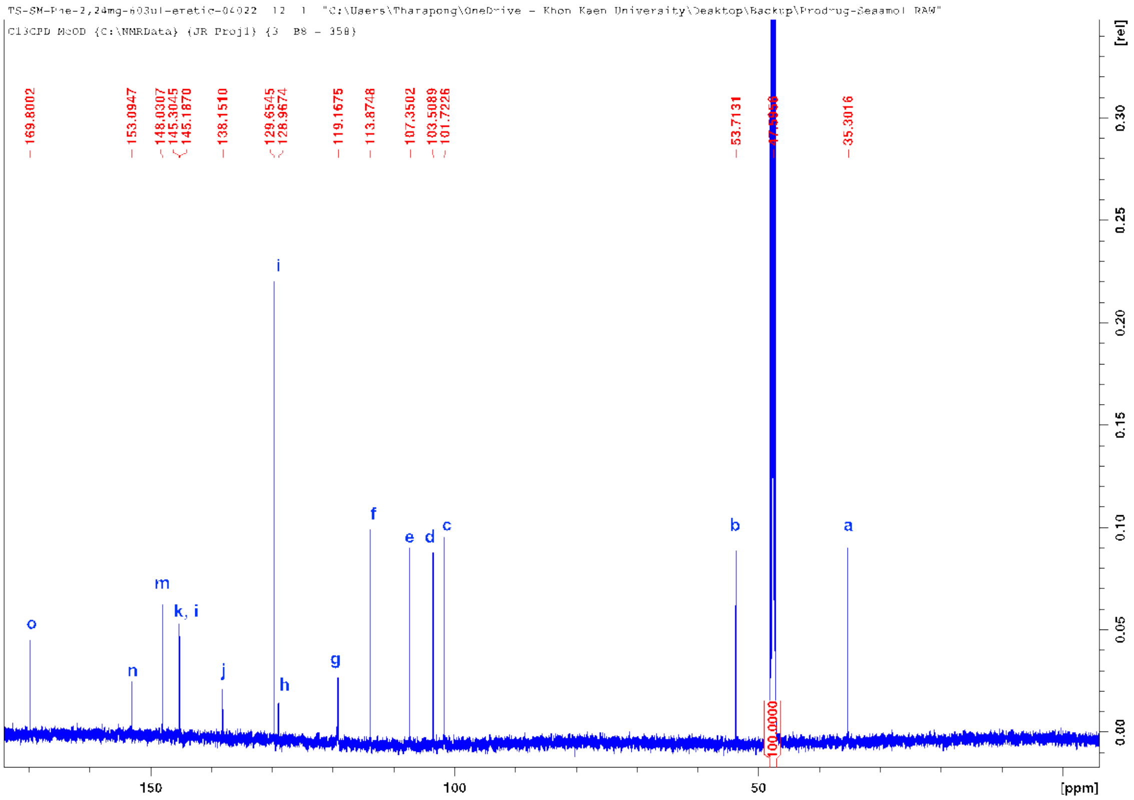

Figure S3. Carbon (^{13}C-NMR) of sesamol prodrug chemical structure. The alphabet **a-o** are carbon positions from the chemical structure from Figure S1.

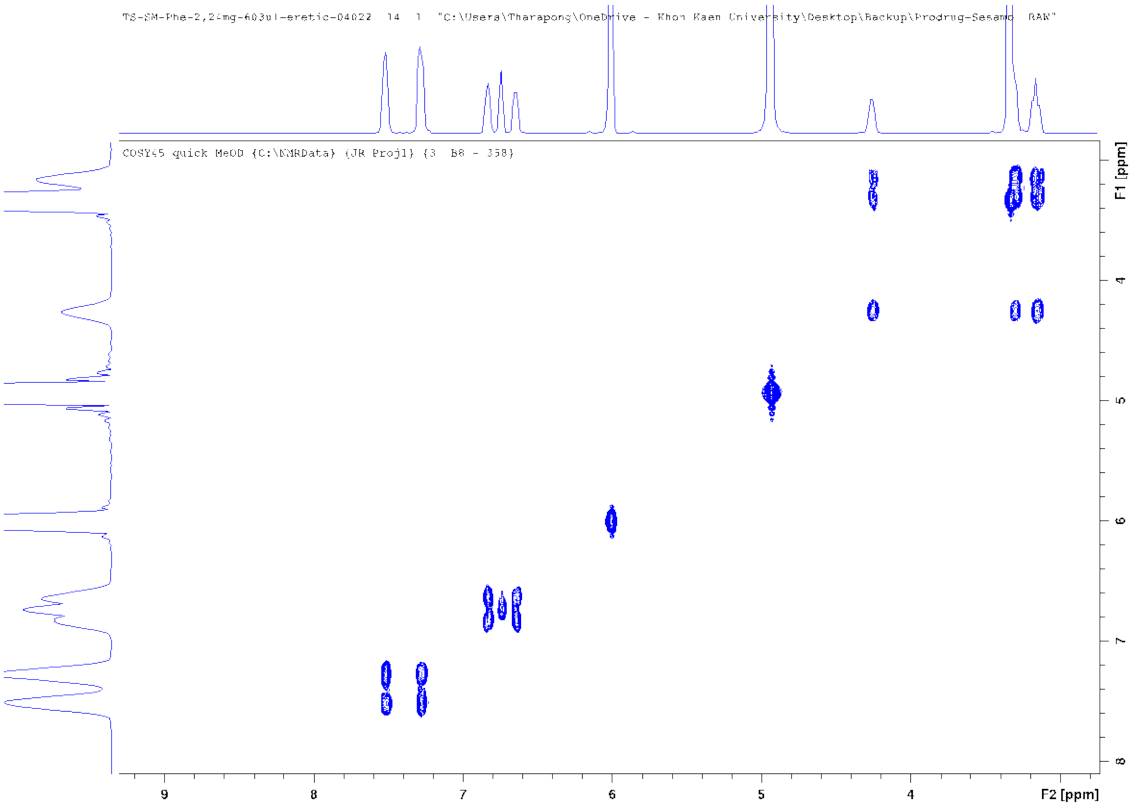

Figure S4. ^{1}H-^{1}H Correlated spectroscopy (COSY) NMR of sesamol prodrug.

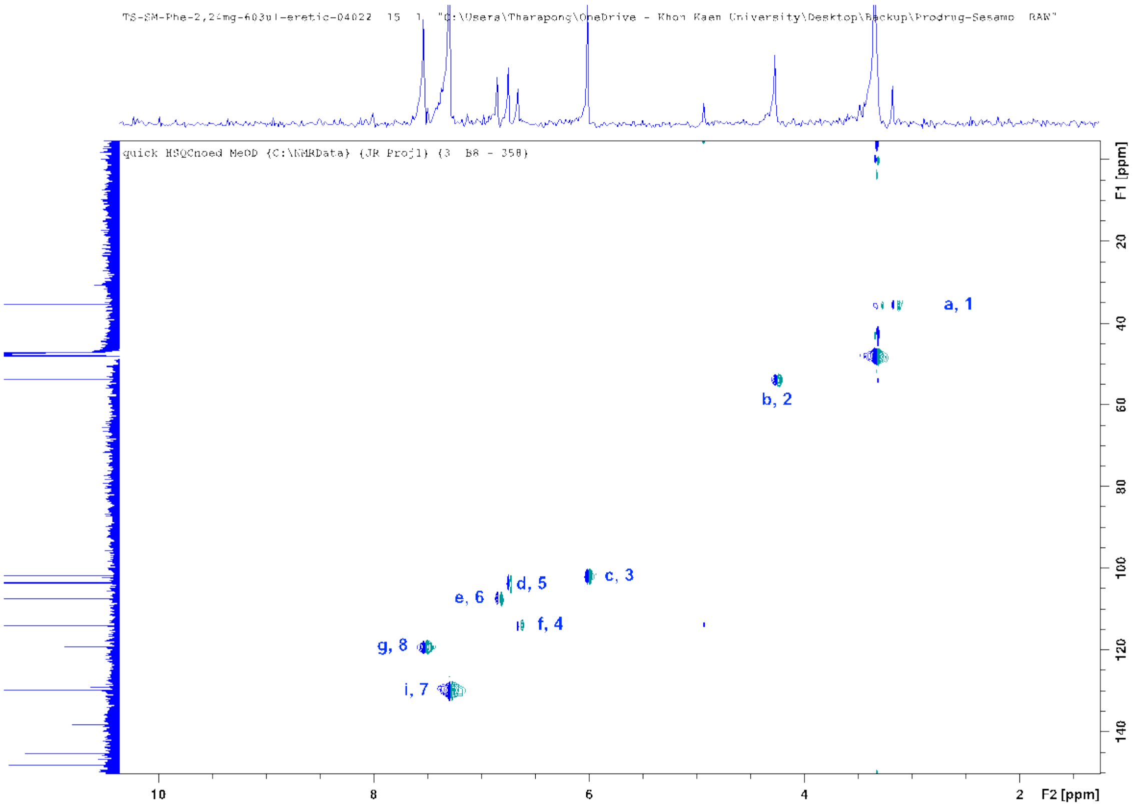

Figure 5S ^{1}H-^{13}C Heteronuclear single quantum coherence (HSQC) NMR of sesamol prodrug. The number and alphabet are related to the proton position and carbon position from the chemical structure from Figure S1.

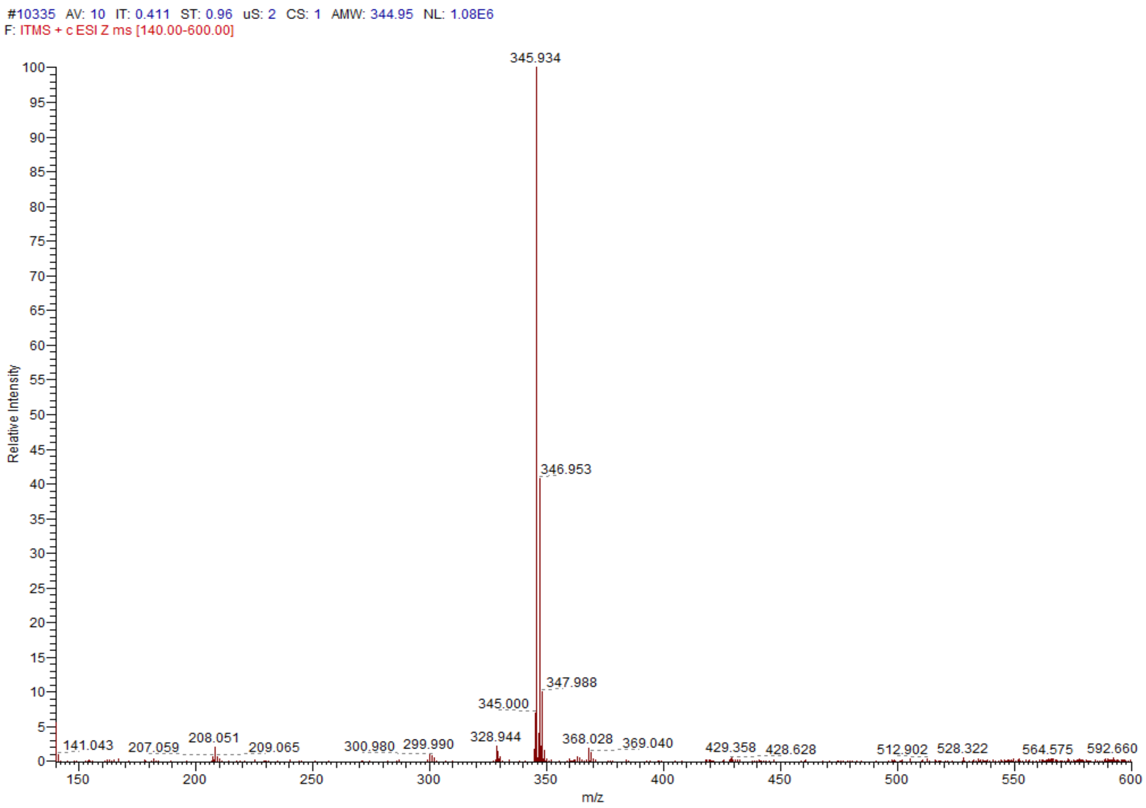

Figure 6S. ESI-MS-positive mode of sesamol prodrug. The base m/z peak is 345.934 m/z. The actual molecular weight of sesamol prodrug is 344.95.
